# Cognitive Impairment and Neurocognitive Profiles in Major Depression—A Clinical Perspective

**DOI:** 10.3389/fpsyt.2022.764374

**Published:** 2022-03-08

**Authors:** Åsa Hammar, Eivind Haga Ronold, Guro Årdal Rekkedal

**Affiliations:** ^1^Department of Biological and Medical Psychology, University of Bergen, Bergen, Norway; ^2^Division of Psychiatry, Haukeland University Hospital, University of Bergen, Bergen, Norway

**Keywords:** MDD, cognitive functioning, scar, trait, state, remission, relapse, residual cognitive symptoms

## Abstract

Increasingly, studies have investigated cognitive functioning from the perspective of acute *state*- to remitted phases of Major Depressive Disorder (MDD). Some cognitive deficits observed in the symptomatic phase persist in remission as *traits* or *scars*. The etiological origin and clinical consequences of the neurocognitive profiles reported in the literature are still unclear and may vary across populations. Deficits are suspected to influence the association between MDD and neurodegenerative disorders and could thus be of particular clinical consequence. The aim of this review is to describe the clinical neuropsychological profile in MDD and how it is related to research during the past decade on cognitive deficits in MDD from a state, trait, and scar perspective. This review, with a clinical perspective, investigates research from the past decade regarding cognitive functioning in MDD in a long-term perspective. We focus on the clinical manifestation of deficits, and the potential neurodegenerative consequences of the neurocognitive profile in MDD. Searches in Medline, PsycINFO and Embase were conducted targeting articles published between 2010 and 2020. Examination of the evidence for long-lasting neurocognitive deficits in major depression within the cognitive domains of Memory, Executive Functions, Attention, and Processing Speed was conducted and was interpreted in the context of the State, Scar and Trait hypotheses. Defining the neurocognitive profiles in MDD will have consequences for personalized evaluation and treatment of residual cognitive symptoms, and etiological understanding of mood disorders, and treatments could potentially reduce or delay the development of neurodegenerative disorders.

## Introduction

Cognitive deficits are a central component in Major Depressive Disorder (MDD) (1–3). It is estimated that 25–70% of the patients will suffer from cognitive deficits (4, 5), however these numbers vary depending on clinical factors such as symptom severity, duration, onset, treatment factors as well as methodological approaches for measuring cognitive functions (4, 6, 7). Thus, there is considerable complexity when it comes to understanding cognitive deficits in MDD in the current literature.

It is clear that cognitive impairment in depression is a substantial problem associated with severe difficulties in occupational, social, and interpersonal functioning (8–10). In addition, deficits are also associated with significantly lower quality of life (11), even in phases of recovery (12, 13). A growing pool of literature during the past decade shows that impairment in cognitive functioning persists in remission and worsens over time with repeated episodes (14), and age (15). Given the wealth of studies showing cognitive deficits in MDD, in addition to important clinical consequences of this disorder, it is important to draw on the literature for potential novel etiological and clinical implications, to prevent and remediate cognitive decline.

**Figure 1:**
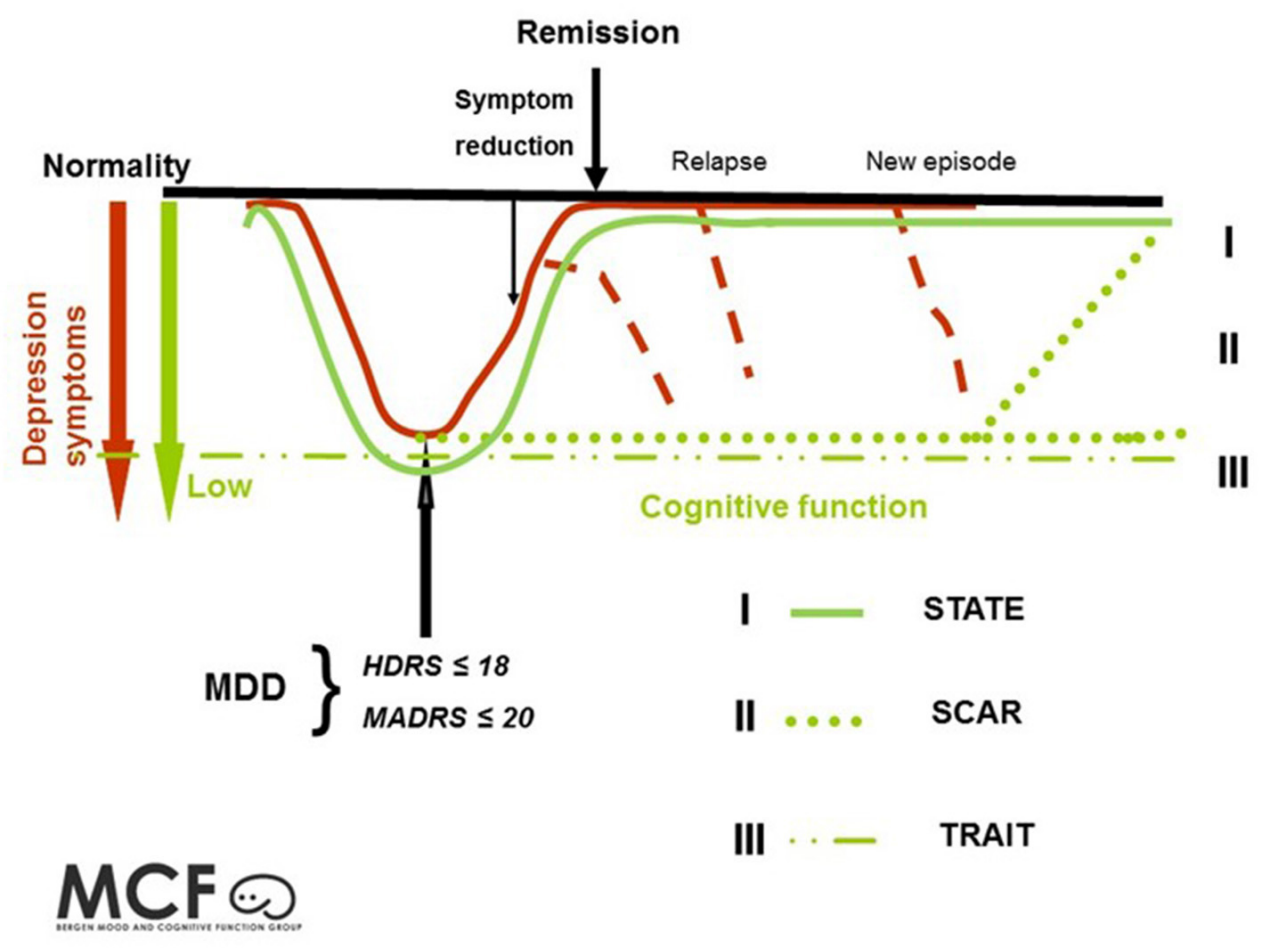
Illustrates three hypotheses regarding the neurocognitive profile in depression. **(I)** The state hypothesis: cognitive impairment is state dependent and follows mood symptoms. See "I". **(II)** The scar hypothesis: cognitive impairment is a result of a scarring effect from the neurotoxic effects of depression. See "II". **(III)** The trait hypothesis: cognitive impairment is related to stable persistent features. See "III". This figure is adapted from Hammar and Årdal (2) and Frank et al. (22).

The causes and consequences of neurocognitive impairment in depression is still debated. Several authors have explored this issue through the state, trait and scar hypotheses (2, 16–19). These hypotheses are essential to understanding the neurocognitive profile in mood disorders because they entail specific hypotheses regarding the etiological development and clinical consequences of cognitive deficits in MDD. The state hypothesis explains the cognitive deficits as caused by the depressive symptom state. This perspective predicts that cognitive impairment will normalize in parallel with affective symptom reduction. The scar hypothesis suggests that depression is neurotoxic and causes irreversible cognitive impairment over time (20, 21). Finally, the trait hypothesis suggests a neurocognitive vulnerability existing prior to the depressive symptoms and claims that cognitive impairment contributes to an increased risk of developing depression, in addition to persistence in remission, representing a risk of relapse. See Figure 1 for further description/discussion.

Among other things, the severity of symptom load in depression will be associated with severity in cognitive impairment, according to the state hypothesis. Following this, neurocognitive impairment is a consequence of clinical symptoms of depression, such as dysphoric mood, reduced motivation, indecisiveness, sleeping problems, loss of energy and a feeling of hopelessness and attentional burden due to worry and rumination. The origin/cause of cognitive impairment is temporary and is caused either by the depression having a transient neurobiological impact, or indirectly, by the depressive symptoms leading to lack of motivation and effort affecting cognitive performance, and/or attentional taxing *via* symptoms such as rumination. Most likely, these three explanations together contribute toward the understanding of the origin of cognitive impairment during depression. However, traditionally the cognitive profile was expected to normalize during symptom improvement, and consequently patients were expected to function on a pre-morbid level in recovery, and not differently than a demographically comparable, non-depressed population. The past decade of research casts doubt over these expectations (14).

The scar hypothesis indicates a progressive decline in cognitive impairment related to duration and number of episodes. In this context, depressions is understood as neurotoxic, causing cognitive impairment (23). Dysregulation of the HPA-axis has been suggested as one of several neurobiological origins interfering with neurogenesis (24–26). Due to the neurogenesis in the absence of depressive episodes, one might expect a possible normalization of cognitive functioning over time. See II in Figure 1. Importantly, this perspective could have implications for the development of neurodegenerative disorders like Alzheimer's disease (23).

The trait hypothesis suggests that a neurocognitive vulnerability (traits) contributes to an increased risk of developing depression. In this perspective, the origin is found in predispositions, prior to illness and independent of clinical state. The origin may be biological, either inheritable and/or caused by environmental mechanisms such as prenatal or early childhood life stress. With this perspective, the cognitive profile is stable over time; thus, the cognitive impairment will not fluctuate with clinical state and persist in remission.

One aspect underlining the importance of understanding neurocognitive impairment is the substantial risk of relapse and recurrence in depression. Even with effective treatments for reducing symptoms of depression, most patients will experience relapse or recurrent episodes (27, 28). While only a few studies have explored the role of residual cognitive symptoms in relapse and recurrence risk, an association has been indicated between cognitive functioning and relapse risk (29, 30). In addition, many patients report substantial cognitive difficulties in everyday life, and it has been shown that subjective cognitive dysfunction is related to functional disability (31), persistence in remission (32), and even predictive of new episodes of MDD (33).

Over the past decade, substantial parts of the research have changed focus from the acute/symptomatic- to the remitted state, and a growing body of literature has focused on the long-term course of this impairment, resulting in heterogenous findings and conclusions [for reviews and meta studies see (10, 14, 18, 21, 34)], with regard to how cognitive deficits are understood.

The aim of this paper is to review the literature from the past decade regarding cognitive functioning in depression, to clarify the role and origin of the long-term neurocognitive profile in depression through the clinical-, and the state, trait, and scar perspective. Furthermore, clinical implications of cognitive residual symptoms, potential increased risk for neurodegenerative disorders, and potential preventive interventions for cognitive enhancement, and suggestions for future studies are discussed.

## Methods

This review is based on computerized searches in Medline, PsycINFO and Embase, exclusively for articles published during the decade between 2010 and 2020 using the terms DEPRESSIVE/ MAJOR DEPRESSION, COGNITIVE DYSFUNCTION, NEUROCOGNITIVE, LONGLASTING, PREVAILED, RESIDUAL, EUTHYMIC, REMISSION in combination. In addition, reference lists were examined for further relevant studies. Every unique abstract, a total of 414, was examined to determine if it is relevant for the topic. Seventy papers were finally included in the summary. Both longitudinal- and cross-sectional original studies were considered relevant, and both reviews and meta-studies were included.

### Cognitive Functioning as a Theoretical Concept

Cognitive functioning is a complex theoretical concept, and there is a lack of consensus concerning the definition and use of the term in the literature (7, 34, 35). One consequence of this is the variety of interpretations and conclusions regarding cognitive functioning in depression.

**Figure 2:**
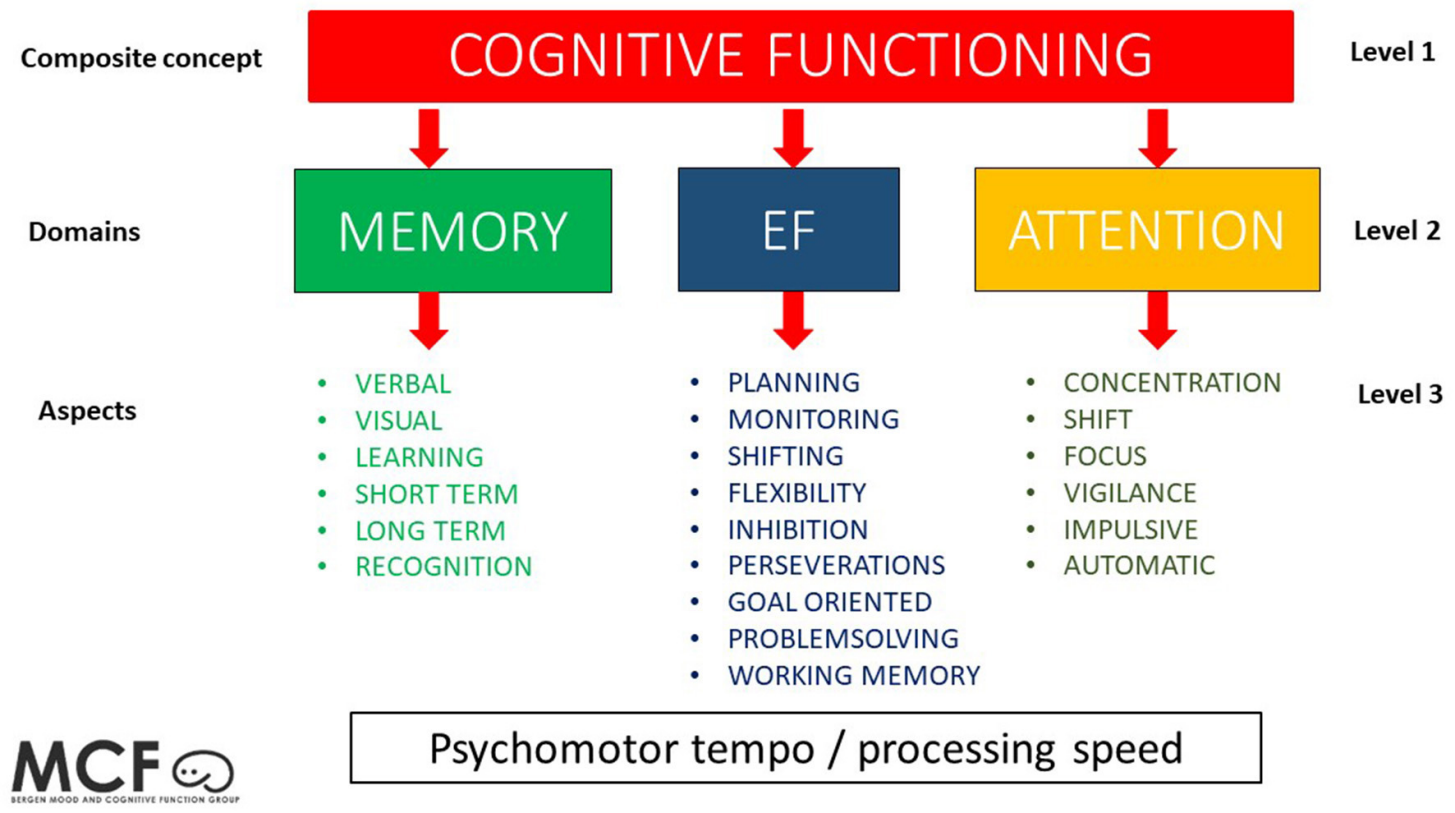
This figure shows a conceptual model of the levels of cognitive functioning, with cognitive functioning as a composite concept (level 1) having three major sub-concepts as domains (level 2) and several specific aspects within each domain (level 3). In this model, psychomotor tempo/processing speed is illustrated as interrelated with all the other domains.

This complexity is sometimes hard to grasp for clinicians and patients outside the field of clinical neuropsychology. As shown in Figure 2, cognitive functioning is a concept consisting of several interrelated sub-concepts defined as domains. Furthermore, within each domain there are several specific aspects. In a traditional neuropsychological assessment, each specific aspect is measured by standardized tests or experimental paradigms. These findings will normally be interpreted and explained on an aspect level in a clinical setting (see Figure 2); however, the literature traditionally describes findings on a domain level, with the risk that important findings on an aspect level will be ignored. When studies report results as composite scores, summarized by scores of different aspects within a specific domain (36, 37), it may lead to a wrong conclusion regarding lack of differences between groups (type 2 error) with the risk that specific impairments will disappear in the composite score. This is of particular importance in clinical neuropsychological work in which the whole ideographical profile is of importance for the individual patient. Consequently, an incorrect portrayal of patients' cognitive profile could be reported, failing to mirror actual challenges in daily life functioning. In addition, using population norms to explain test results (38) may ignore the important ideographic interpretation of how the different cognitive tests are related to the individual patient. In addition, some norms are not standardized for regional conditions and could thus underestimate deficits (39). In addition, a recent meta study by Parkinson et al. (40) argued that results in one domain may not reflect the effects on one test, thus precluding results from single tests.

Composite scores, however, are important in research to identify latent variables representing different cognitive domains, avoiding task impurity problems, and useful for structural equation models investigating cognitive functions above and beyond the clinical setting (7).

With this in mind, the literature from the past decade will be summarized with a focus on cognitive functioning over time, in a long-term perspective, investigating the three previously mentioned hypotheses in particular, in the cognitive domains of memory, executive functioning, attention, and psychomotor processing speed. The concept long-term perspective is used to describe the cognitive profile over time in non-symptomatic phases, which can be reflected in both cross- sectional and longitudinal studies.

## Results

### The Domain of Memory

Patients with a history of depression often report that they experience memory problems, both in an acute phase (41) and in a recovery phase (42). Based on clinical practice, patients not only describe this problem evidenced in themselves, but also in family members and others. This could lead to increased stress, frustration, relational problems, and negative self-representation (43). Many former patients relate these memory problems to possible brain damage or dementia, leading to a negative impact on self-representation, rumination and coping. Such an interpretation combined with the lack of correct knowledge regarding the role and origin of residual cognitive impairment might lead to an increased risk of relapse and new episodes.

Numerous studies have investigated aspects of memory during the past decade and findings so far appear divergent and non-conclusive. Lee et al. (44) concluded in their meta-study that memory represented a state marker being associated with clinical state in first episode patients. This was also evident in the meta-study by Ahern and Semkovska (45), concluding that first-episode patients showed normalized learning, memory, and autobiographic memory in remission. Pu et al. (46) found deficits in verbal memory but only for a subgroup of patients with MDD. This group also showed deficits in information processing that could influence memory consolidation. This was also the case in Lee et al. (44), where patients with mild depressive symptoms showed second largest impairments in verbal memory, only superseded by processing speed. An older MDD group showed deficits in visual and verbal, learning and memory, but also in most cognitive functions. Xu et al. (47) reported immediate visual memory impairment (WMS-R Immediate Visual Reproduction; copying figure after 10 s exposure) in patients in the depressed state and in remission compared to healthy controls, suggesting visual memory deficits may be a trait for mood disorders. This finding, however, was not supported in a study conducted by Hammar and Schmid (48), whose findings indicate that visual memory performance in patients with major depression normalizes during a 9-month period, thus indicating a state-related relationship. This study had a small sample, suggested a deficit in copying at follow up, and lacked the immediate condition that could be most analogous to Xu et al. (47). Thus, a trait/scar deficit in visual construction is indicated. Hammar et al. (49) suggested that MDD patients showed intact verbal memory performance, a deficit in immediate verbal learning, and deficits in visual memory in the symptomatic phase. Immediate verbal learning deficits were still evident during partial remission (50). Shimizu et al. (51) reported that remitted MDD patients had poorer verbal memory, both immediate and delayed (as well as deficits in most other cognitive areas), compared to healthy controls. This was in line with findings showing that despite significant improvement in memory from the symptomatic phase to remission, patients were still significantly impaired compared to healthy controls (52). Wekking et al. (53) found impairment in several measures of memory in remitted patients but could not establish that the impairment predicted future relapse within in a 24-month period. Vasavada et al. (54) found deficits in verbal learning before ECT treatment, but also following symptom reduction, suggestive of trait or scar effects. A study of remitted patients and their high-risk twins (55) did not find any verbal memory impairments in either group, contrary to what would be expected from a trait perspective. However, a recent meta-study found small impairments in most cognitive functions in first-degree relatives of patients with MDD, including in memory, supporting this hypothesis (56). Finally, studies investigating first-episode patients and patients with recurrent depression with biological correlates have supported a scar hypothesis: Hansson et al. (25) reported no relationship between an abnormal HPA axis and cognitive dysfunction in verbal memory in first-episode patients. However, abnormal HPA-axis and cognitive impairment was evident in patients with a history of recurrent depression, indicating that the episodes might have a scarring effect on verbal memory functioning (24). Tully (57) also finds potential scarring effects, with depressive symptoms and high blood pressure associated with decline in visual memory. This view is also supported by a review by Mcintyre et al. (58), that posits that a subgroup of individuals with MDD show progressive decline in memory. A study on late-life depression suggested that depressive symptoms were a prodrome for Alzheimer (59), that could suggest a dementia state effect on reduced memory in late life MDD (see Table 1).

**Table 1:** Findings within the domain of memory regarding origin of impairment.

**Study**	***N***	**Age (SD)**	**Sex**	**Education (SD)**	**Depression severity (SD)**	**Number of episodes**	**Study design**	**Neuropsychological tests**	**Key outcomes**
The state hypothesis									
Lee et al. (44)	15 samples with 644 patients	39(10)	Not reported	Not reported	Not reported	First episode patients	Meta-analysis	Logical Memory 1 and 2, Rey Auditory Verbal Learning Test (RAVLT), California Verbal Learning Test (CVLT-II), Hopkins Verbal Learning Test (HVLT), Buschke's Selective Reminding Test (SRT). Visual Reproduction 1 and 2, Rey Complex Figure Test (RCFT), Weschler Memory Scale (WMS)	Memory functioning was associated with clinical state
Hammar and Schmid (48)	Baseline: 24 MDD patients (PG) 24 individually matched healthy controls (HC)	PG: 38(11) HC: 38(11)	18 females	PG:12(2) HC: 13(2)	T1: HDRS 23(5) T2: HDRS 11(5)	Recurrent depression minimum 2 episodes	Longitudinal with baseline (T1) and 9 months follow up (T2)	Rey Complex Figure Test	Significant improvement in depressions symptoms and in visual memory impairment
Ahern and Semkovska (45)	31 studies with 994 patients	Weighted mean age: Patient 27 Control 30	patients: 586 females Control: 761 females	Not reported	Not reported	First Episode patients	Review and meta-analysis	Several test in domains of: Autobiographical memory Visual learning and memory Learning Delayed memory Verbal learning and memory Recognition Learning Delayed memory	Remission was associated with a normalization of function in, learning and memory, autobiographical memory
Pu et al. (46)	170 patients with non-psychotic MDD	38(12)	79 females	Duration of education 15(2)	HAMD: 8(4)	Not reported Duration of illness 8(6) years	Cross-sectional	Brief Assessment of Cognition in Schizophrenia (BACS) Verbal memory: List Learning Test	Impaired memory was associated with the clinical state of MDD
Javaherian et al. (59)	Depressive symptoms (DS) *n* = 54 No Depressive symptoms (NoDS) *n* = 300	DS = 71(5) No DS 72 (5)	DS = 38 females No DS = 151 female,	DS = 15(3) No NoDS = 16 (3)	GDS: 3(2) NPI-Q item 5a (=yes)	Not reported	Cross-sectional	Free and Cued Selective Reminding Test, the Associate Learning subtest from the Weschler Memory scale (WMS), WMS-Revised Logical Memory	Depressive symptoms was associated with reduced episodic memory in later stage preclinical Alzheimer's
The scar hypothesis									
Hansson et al. (24)	24 MDD patients (PG) 24 individually matched healthy controls (HC)	PG: 38(11) HC: 37(11)	18 females	PG:12(2) HC: 13 (2)	MADRS 27(5)	Recurrent depression minimum 2 episodes	Cross sectional	California Verbal Learning Test (CVLT-II) Rey Complex Figure Test	Findings indicate that dysregulation of the HPA-axis is related to poor verbal memory functioning
Hansson et al. (25)	21 MDD patients (PG) 21 individually matched healthy controls (HC)	PG: 26(6) HC: 25(6)	12 females	PG: 14(2) HC: 14 (1)	MADRS: 24(4)	First episode MDD patients (FE).	Cross sectional	California Verbal Learning Test (CVLT-II) Rey Complex Figure Test	No associations between cortisol levels and cognitive functioning, indicating that FE patients are not as affected as recurrent MDD patients.
Vasavada et al. (54)	44 MDD 33 demographically similar controls (CG)	MDD: 41 (13) CG: 39 (12)	MDD: 26 females CG: 19 females	MDD: 16 (3) CG: 17 (2)	M ADRS T1 = 37 (8) T4 = 17 (12)	>1 Episode, 16 years mean duration	Longitudenal	Hopkins Verbal Learning Test—Revised, Brief Visuo- spatial Memory Test—Revised	Verbal learning deficits initially, and no significant improvement in symptom remission
Semkovska et al. (14)	11 882 major depressive episode remitters 8,533 healthy controls	Not reported specifically	Not reported specifically	Not reported specifically	Not reported specifically	Not reported specifically	Systematic review and meta-analysis	Several tests in domain measures of Verbal memory and visuo or spatial memory	Deficits in long-term memory persist in remission from a major depressive episode and worsen with repeated episodes
The trait hypothesis									
Xu et al. (47)	293 Unipolar depression patients (UP) 202 Healthy Controls (HC)	UP: 35(13) HC:34(10)	162 females	UP: 11 (4) HC: 13 (4)	HDRS: 27(6)	2(2)	Longitudinal Baseline and 6 weeks follow up	Immediate Visual Reproduction of Wechsler Memory Scale-Revised in China (WMS-RC)	Remitted unipolar patients showed cognitive impairment in executive function in addition to processing speed and visual memory
Mackenzie et al. (56)	3,246 First-degree relatives MDD (fdrMDD) 5,222 controls	fdrMDD 15(14) controls 15(12)	1,872 femalesfdrMDD 2,921 female controls	Not reported	Not reported	Not reported	Systematic review and Meta analysis	CVLT, RAVLT, Verbal Paired Associates Initial and Delay Recall RCFT Self-Referential Encoding and Incidental Recall Task, Autobiographical Memory Test, Computerized Autobiographical Memory Test	Globally impaired cognition in fdrMDD, including for the domain of memory
Tully et al. (57)	2,812 Older participants divided by: late onset symptomatic (*n* = 105) asymptomatic (*n* = 200) early onset smptomatic (*n* = 51) asymptomatic (*n* = 74)	Median age 72	1,788 females	Not reported	Not reported	Not repored	Prospective cohort study	Visual memory (BVRT)	Late onset MDD showed global decline, and in visual memory with interactions between MDD and white matter intensities
McIntyre et al. (58)	Not reported	Not reported	Not reported	Not reported	Not reported	Not reported	Review	Not reported	A subset of adults MDD patients show progressive decline in memory

*MADRS, Montgomery-Åsberg Depression Rating Scale*.

In conclusion, the literature supports evidence of a long-term memory impairment in depression. This is not independent of attentional and learning deficits, as evidenced by the sustained difficulties with immediate memory, and it could therefore be influenced by impaired informational encoding more than a long-term memory deficit. In addition, all three hypotheses concerning role and origin have partial support. Hence, the neurocognitive memory profile in depression is neither specific nor conclusive and requires a multidimensional approach. This domain is of particular interest with regard to development of neurodegenerative disorders and is often affected first in the development of Alzheimer's disease. Studies seem to find increased deficits in memory related to depression with increasing age (14, 60).

### The Domain of Executive Functioning (EF)

Patients who have experienced or are experiencing depression frequently report that they often have difficulty performing tasks that require initiating or finishing activities, problem solving or getting an overview of situations, multitasking, or emotional regulation; inhibiting negative or troubling thoughts (41–43). We clinically relate these tasks to a higher order of functioning for regulation of behavior, thoughts and emotions –defined as Executive Functions.

Aspects of EF have been investigated in several studies, and findings tend to highlight inhibition, defined as suppressing an automatic response in order to make a less automatic but task-relevant response (61). The concept of inhibition has been operationalized differently in different studies depending on the neuropsychological task used (7). Lee et al. (44) suggested in his review that inhibition could be a trait marker in first-episode patients. This conclusion was supported in findings from several studies conducted by Schmid and Hammar (62), who stated that impaired inhibition on the stroop test, in addition to semantic fluency, is present early in the course of MDD, indicating that EF represents a trait in MDD, irrespective of symptom severity and number of previous episodes. Moreover, the authors showed that impairment in inhibition and switching and semantic fluency in first-episode MDD persisted in long-term follow up (63), with the former associated with relapse during the first year after the first episode (30), and with deficits in inhibition in a subgroup with relapse 5 years later (64), suggesting a relationship between impaired ability in EF of inhibition and switching and relapse in MDD. These findings were also evidenced in patients with recurrent depression and showed that impaired inhibition in the acute phase persisting in phases of symptom reduction (65). Another study on the same patient group showed that impaired inhibition in the symptomatic phase was strongly correlated with impaired inhibition in long-term follow up, indicating that this may represent stable a trait marker in recurrent MDD (66). Moreover, one of the longest follow-ups investigating the same recurrent MDD patients and controls over a 10-year period showed that patients were still impaired with regard to inhibition (13). A twin study did found no EF deficits, neither affected- nor high-risk twins (55). However, a meta study of cognition in first-degree family members did find small effects for deficits in EF (56). This strengthens the hypothesis of at least some trait relation. In addition, findings regarding neural correlates and impaired inhibition were reported in partially remitted and remitted-recurrent MDD patients showing hypoactivation in striatal areas (67). These findings could be interpreted as results of scars or being trait-related. This is in line with findings from Peters et al. (19), who concluded that impaired inhibition as cognitive control in acute and remitted states may represent a trait vulnerability or an early course scar of MDD viable target for secondary prevention or cognitive remediation. Also, Bora et al. (60) concluded in their meta-analysis that response inhibition seems to be a persistent feature in adult-onset MDD, thus supporting the trait hypothesis. They reported that among all cognitive functions, inhibitory control showed the largest magnitude of observed deficits in euthymic MDD patients compared to controls. However, in contrast to all previous findings, Aker et al. (68) reported no deficits in cognitive inhibition in remitted patients, only in response inhibition. Wekking et al. (53) also found persisting deficits in most cognitive measures, except inhibition. Both studies used a contrast score that only approached statistical significance and results similar to other studies (30, 63).

Other studies have focused on different aspects of EF. Contrary to several conclusions regarding trait-related explanations, Roca et al. (69) showed normalization in several cognitive measures such as problem-solving in first-episode and recurrent-episode remitted patients; however, they did not find improved inhibition in the sample. Still, they conclude that remission, rather than numbers of previous episodes, has a high impact on cognitive performance in MDD patients, thereby supporting a state model. Other studies consistently find EF impaired in the depressed state (70, 71), with some inconsistencies with regards to improvements with symptom reduction (54, 72, 73). Pu et al. (46) found small correlations between an EF composite and depressive symptoms; however, this could vary by specific EF tasks measured (63). Age could also influence EF deficits, Boedeker et al. (74) found impairments in switching, but no statistically significant differences in inhibition, in an aging MDD sample. Maalouf et al. (75) using a planning task in adolescents with acute and remitted MDD and they stated that planning and impulsivity appear to be state-specific markers of MDD in adolescents, and are related to depression severity and are not persistent in remission, but a relatively small- and poorly matched sample as well as tasks used could explain this. Early experiences could influence cognition, and Saleh et al. (76) found worse EF in a MDD group with early life stress. Chakrabarty et al. (77) found that only a MDD population with trauma showed persisting deficits in WM in remission. Albert et al. (36) found a relationship between longer duration of depression age, and EF with no effects of current depression severity on performance. The authors concluded that cognitive performance worsens with recurrence over the life span. These findings can be interpreted as support for the scarring hypothesis. Bhardwaj et al. (78) drew the same conclusion, demonstrating an impairment in a planning and problem-solving task in recovered MDD patients and found that performance was correlated with number of previous episodes of depression. They concluded that impairments of EF are present in recovery and are thus not simply state markers, but instead scars caused by previous episodes (see Table 2).

**Table 2:** Findings within the domain of executive functions regarding origin of impairment.

**Study**	***N***	**Age (SD)**	**Sex**	**Education**	**Depression severity**	**Number of episodes**	**Study design**	**Neuropsychological tests**	**Key outcomes**
The state hypothesis									
Maalouf et al. (75)	20 adolescents with MDD in acute episode (MDDa) 20 previously depressed adolescents in remission (MDDr) 17 healthy control participants (HC)	MDDa: 15 (2) MDDr: 15(1) HC: 15 (2)	MDDa: 17 females MDDr: 15 females HC: 9 females	Not reported	CDRS-R MDDa: 59 (11) MDDr: 2 (3) HC:19 (2)	MDDa: 1.4 (0.6) MDDr: 1.2 (0.5)	Cross-sectional	The Cambridge Neuropsychological Tests Automated Battery (CANTAB): (a) Stockings of Cambridge (SOC) task, as a measure of executive function; (b) Rapid Visual Processing (RVP) task, as a measure of sustained attention; and (c) the Delayed matching to Sample task (DMS), a measure of visual short-term memory	Executive dysfunction and impulsivity appear to be state-specific markers of MDD in adolescents that are related to depression severity and not present in remission
Roca et al. (69)	26 First episode (FE) 53 recurrent episode (RE) depressive patients	FE: 44 (9) RE: 47 (8)	FE: 21 females RE: 41 females	University degree FE: 19% RE: 13%	HDRS FE: 22 (3) RE:24 (5)	RE: 4 (3)	Observational longitudinal cohort study	TMT AoB Digit Span Stroop Tower of London Verbal Fluency task (FAS) Semantic Verbal fluency (animals)	Show normalization in several cognitive processes, such as problem solving, however not in inhibition
Pu et al. (46)	170 patients with non-psychotic MDD	38(12)	79 females	Duration of education 15(2)	HAMD: 8 (4)	Not reported Duration of illness 8 (6) years	Cross-sectional	BACS	Three MDD subgroups, one with global impairments including executive dysfunction
Mak et al. (71)	35 MDD, 35 Healthy matched controls (hMC)	MDD: 25 (4) hMC: 22(3.)	20 females MDD 23 females hMC	MDD: 14 (2) hMC: 15.4 (1.22)	MADRS MDD: 23 (5)	1 (1)	Cross sectional case-control	WCST, TMT, VFT	MDD scored worse than hMC on excecutive functions (WCST) and TMT B (n.s. medium e.s.)
Koo et al. (70)	20 MDD, 20 Healthy controls (HC)	MDD: 51 (11) HC: 76 (6)	11 females MDD 13 females HC	Not reported	BDI MDD: 28 (8) HC: 3 (3)	3 (2)	Cross sectional case-control	TMT B, Stroop	MDD showed poorer performance than HC across all cognitive tests, including TMT B and Stroop interference
Boedeker et al. (74)	30 MDD, 90 Healthy controls (HC)	MDD: 74 (4) HC: 47 (13)	22 females MDD 47 females HC	MDD: 9 (2) HC: 9 (1)	Not reported	Not reported	Cross-sectional	Verbal fluency, TMT, Stroop	MDD showed poorer performance than HC on TMT B, Verbal fluency, and Stroop (n.s.)
The scar hypothesis									
Bhardwaj et al. (78)	20 patients in recovery from recurrent unipolar (PG) depression 20 healthy controls (HC)	PG: 34 (8) HC: 33 (8)	PG: 2 females HC: 3 females	PG: 13 (3) HC: 13 (3)	HDRS: 4 (2)	4 (2)	Cross-sectional	WCST	Cognitive impairment correlated with numbers of previous episodes
Xu et al. (47)	293 Unipolar depression patients (UP) 202 Healthy Controls (HC)	UP: 35 (13) HC:34 (10)	131 males and 162 females	UP: 11 (4) HC: 13 (4)	HDRS: 27 (6)	2 (2)	Longitudinal Baseline and 6 weeks follow up	Modified WCST-M Tower of Hanoi (TOH) Trail Making Test-part B (TMT-B)	Remitted unipolar patients showed cognitive impairment in executive function
Hammar et al. (67)	17 partially remitted and remitted MDD patients (PG) 17 Healthy Controls (HC)	PG: 41(11) HC: 40(13)	PG: 3 males and 13 females HC: 3 males and 14 females	Not reported	HDRS: 7 (7)	At least 2 previous episodes	Cross-sectional	Experimental paradigm with a combination of a Stroop task and a n-back task	Striatal hypoactivation and impaired cognitive performance in a sample of partially remitted MDD patients compared to never-depressed controls, indicating neuronal scarring from the disorder
Albert et al. (36)	91 depressed (PG) 105 non-depressed (HC)	PG: 36 (9) HC:30 (9)	PG: 30 males 61 females HC: 37 males and 68 females	PG: 15 (2) HC: 16 (2)	MADRS: 24(4)	Mean Duration in days: 2116 (1800)	Cross-sectional	Executive function: COWAT, Trail Making B time semantic fluency Stroop Color-Word interference condition	A relationship between longer duration of depression age, and EF with no effects of current depression severity on performance
Saleh et al. (76)	64 antidepressant free depressed (PG) 65 non depressed (CG)	PG: 35 (9) CG: 29 (9)	39 females PG 43 females CG	PG: 35.1 (8.9) CG:29 (9.2)	MADRS PG: 25 (5)	Episodes not reported, duration in years 6 (5)	Cross sectional case-control	WM composite consisting of digit span	Found worse WM (but not EF composite) in a MDD group with early life stress
Vasavada et al. (54)	44 MDD 33 demographically similar controls (CG)	MDD: 41 (13) CG: 39 (12)	MDD: 26 females CG: 19 females	MDD: 16 (3) CG: 17 (2)	M ADRS T1 = 37 (8) T4 = 17 (12)	>1 Episode, 16 years mean duration	Longitudinal	Trail Making B, Stroop	Trail Making B poorer in MDD and did not improve following remission
Chakrabarty et al. (77)	MDD without maltreatment (DM+): 93 MDD with maltreatment (DM-): 90 Healthy controls with maltreatment (HM+): 22 Healthy controls without maltreatment (HM-): 80	DM+: 37 (12) DM-: 34 (13) HM+: 34 (10) HM-: 33 (11)	DM+: 63 females DM-: 51 females HM+: 12 females HM-: 54 females	DM+: 14 (2) DM-: 14 (2) HM+: 16 (2) HM-: 16 (2)	MADRS DM+: 31 (6) DM-: 29 (6)	DM+: 4 (4) DM-:3 (3)	Longitudinal with baseline, 8 weeks and 16 weeks follow-up	Central Nervous System Vital Signs (CNS-VS) computerized battery with a global composite score	Maltreatment may be a risk factor for more severe and persistent cognitive deficits in adult MDD
The trait hypothesis									
Lee et al. (44)	15 samples with 644 patients	39 (10)	Not reported	Not reported	Not reported	First episode patients	Meta-analysis	WCST, Modified Card Sorting Test (MCST); CANTAB Intradimensional/Extradimensional-Shift (ID/ED)	Executive Functioning seems to be a trait-marker
Peters et al. (19)	Remitted MDD (rMDD): 62 Healthy controls (HC): 43	rMDD: 21 (2) HC: 21 (2)	47 females rMDD 23 females HC	rMDD: 14 (1) HC: 15 (1)	HAMD-D rMDD: 3 (3)	Not reported	Cross-sectional	Stroop, TMT, COWAT, Go/no-Go	Impaired inhibition as cognitive control in acute and remitted states may represent a trait vulnerability or an early course scar of MDD
Schmid et al. (66)	20 recurrent MDD patients (PG) 19 healthy controls (HC)	PG: 38 (11) HC: 38 (11)	PG: 18 females HC: 18 females	PG: 12 (2) HC:13 (2)	MADRS: 15 (6)	At least 2 previous episodes	Longitudinal with baseline and 9 months follow up	D-KEFS Color–Word Interference Test (CWIT) The D-KEFS Verbal Fluency Test (VFT)	Recurrent MDD patients show a prolonged impairment in inhibition and semantic fluency
Årdal and Hammar (13)	19 recurrent unipolar MDD patients (PG) 19 healthy controls (HC)	Baseline PG: 43(10) HC: 42 (10)	PG: 10 females HC: 10 females	Baseline PG: 14(4) HC: 14 (4)	HDRS: 5	Total numbers of episodes: 10	Longitudinal with Baseline, 6 months and 10 years follow ups	The Stroop test	Long-lasting impairment in cognitive inhibition at the 10-year follow-up study
Schmid and Hammar (62)	30 MDD patients (PG) 30 individually matched healthy controls (HC)	PG: 26(6) HC: 26 (6)	16 males and 14 females	PG: 14 (2) HC: 14 (2)	MADRS: 25(4)	First episode patients	Cross-sectional	D-KEFS Color–Word Interference Test (CWIT) The D-KEFS Verbal Fluency Test (VFT)	Impaired inhibition on the stroop test, in addition to semantic fluency are present early in the course of MDD
Schmid and Hammar (30)	28 First episode MDD patients (PG) 28 healthy controls (HC)	PG: 27(5) HC: 27(5)	PG: 14 females HC: 14 females	PG. 14(2) HC: 15(2)	MADRS: 10(6)	First episode patients	Longitudinal with baseline and 1-year follow up	D-KEFS Color–Word Interference Test (CWIT)	Impaired ability in the EF of inhibition/switching was related to vulnerability for relapse
Bora et al. (60)	27 studies with 895 patients with MDD 993 healthy controls	Specified for each study included	61% females	Specified for each study included	Specified for each study included	Specified for each study included	Meta-analysis	Global composite score by averaging effects sizes.	Poor response inhibition seems to be persistent in adult-onset MDD
Mackenzie et al. (56)	3,246 First-degree relatives MDD (fdrMDD) 5,222 controls	fdrMDD 15 (14) controls 15 (12)	1,872 fe male fdrMDD 2,921 female controls	Not reported	Not reported	Not reported	Systematic review and Meta analysis	WCST, Intra/Extra dimensional Set Shifting, Stroop, TMT B, Digit span, letter number substitution, letter n-back, hot executive functions (various tasks).	Small (*p* = 0.10) e.s. for poorer EF in first degree relatives of patients with MDD suggestive of genetic defecits in EF
Ji et al. (72)	67 patients with MDD (MDD) 56 Healthy controls (CG)	MDD: 31 (10) CG: 34 (13)	MDD: 37 females CG: 31 females	MDD: 14 (3) CG: 13 (5)	HAMD-17: MDD 21 (3) CG: 2( 2)	4 (2)	Longitudinal with a 6 month follow up	Digital symbol substitution, and digit span forwards- and backwards test	Persisting deficits in WM in remission
Ronold et al. (63)	23 MDD patients (PG) 20 matched healthy controls (HC)	MDD: 31 (6) HC: 30 (6)	MDD: 12 females CG: 11 females	MDD: 15 (2) HC: 17 (2)	MADRS: 9 (8)	Not reported	Longitudinal five year follow up of first episode MDD	D-KEFS: CWIT, VFT, TMT	Persisting deficits in inhibition unrelated to depressive symptoms

*COWAT, Controlled Oral Word Association Test; BDI, Becks Depression Inventory; WCST, Wisconsin Card Sorting Test; TMT, Trail Making Test; CDRS-R, Children's Depression rating Scale-revised; MADRS, Montgomery-Åsberg Depression Rating Scale*.

In sum, existing research supports the assumption of a long-term impairment within the EF domain in general, evident in inhibition in particular, with evidence indicating a trait-related profile. However, all three hypotheses regarding role and origin in EF were supported. Similarly to findings within the memory domain, the neurocognitive profile of EF in depression is neither specific nor conclusive.

### The Domain of Attention

Depressed patients and formerly depressed patients often report problems maintaining attention during conversations or when reading a book or watching TV, etc. These problems have an impact on daily life functioning and may frequently be interpreted as ignorance rather than the result of a cognitive impairment related to the depression or as a residual cognitive symptom (41, 42). This is clinically related to the domain of attention.

Several studies confirm that attention deficits are related to depression both in the acute phase of the illness (2, 44) and in remission (51, 79, 80). Ji et al. (72) found improvements in digit span in remission, that could indicate a relationship between MDD and attention. In addition, another study found an association between attention and inflammatory markers, which could partly explain state effects in attentional deficits. Findings are divergent, however, some studies report no attentional deficits measured by digit span in MDD in patients both in the acute depressive state and in remission (47). Consistent with this, Boedeker et al. (74) did not find deficits in digit span in an aging MDD sample. This could reflect heterogeneity in the neurocognitive profile. Studies that separate subgroups of MDD in first-episode and recurrent-episode showed that first-episode patients differed in neurocognitive profile: While first-episode patients demonstrated no impairment in attention in effortful information processing in the symptomatic phase of depression (81), the group suffering from recurrent episodes showed an impairment in symptomatic and symptom reduction phases, which *normalizes* over a 10-year period (82). Such findings might support the Scar hypothesis, showing that duration could be a critical cue to the attentional deficit. These findings were done with a novel paradigm measuring visual attention and might not be generalizable to all other aspects of attention, however. Differences in age between FE and recurrently depressed could perhaps explain this (83). Pu et al. (46) also found subgroups with differing deficits, with one showing among other cognitive deficits, impaired attention. Clery-Melin and Gorwood (84) found differing outcomes supporting the Trait hypothesis; they showed that attention measured by omission mistakes was an unchanged marker before and after treatment and could predict clinical and functional outcome. They interpreted their findings as reflecting a specific ability to control attention and thereby regulate emotional stimuli, thus representing a trait resilience marker, meaning that patients with enhanced ability in attention are more likely to achieve full clinical and functional remission. Attentional control could arguably be considered an EF, however. In addition, this could illustrate the differences in attentional tasks between error scores and RT. The trait perspective was also supported in a meta-analysis conducted by Lee et al. (44), in which they concluded that attention is more likely a trait-marker in first-episode patients. Attention is a complex cognitive domain and is highly interrelated with the other cognitive domains such as memory, EF and psychomotor tempo and could thus influence all other domains (35) (see Table 3).

**Table 3:** Findings within the domain of attention regarding Origin of Impairment.

**Study**	***N***	**Age (SD)**	**Sex**	**Education**	**Depression severity**	**Number of episodes**	**Study design**	**Neuropsychological tests**	**Key outcomes**
The state hypothesis									
Ye et al. (85)	30 patients with MDD (MDD) 30 Healthy controls (HC)	MDD: 42(11) HC: 42(10)	MDD 18 females HC 17 females	MDD 11(4) HC 12(4)	PHQ-9 ≥ 7	Not reported	Case control	Rapid Visual Information Processing (RVP) from CANTAB	Poorer attention in MDD relative HC, IL-6 levels associated with impaired sustained attention
Pu et al. (46)	170 patients with non-psychotic MDD	38 (12)	79 females	Duration of education 15 (2)	HAMD: 8 (4)	Not reported Duration of illness 8 (6) years	Cross-sectional	BACS	Three MDD subgroups, one with attention impairments
Ji et al. (72)	67 patients with MDD (MDD) 56 Healthy controls (CG)	MDD: 31 (10) CG: 34 (13)	MDD: 37 females CG: 31 females	MDD: 14 (3) CG: 13 (5)	HAMD-17: MDD 21 (3) CG: 2 (2)	4 (2)	Longitudinal with a 6 month follow up	Digital symbol substitution, and digit span forwards- and backwards test	Poorer cognitive functioning in MDD group, remission associated with improved attention
The scar hypothesis									
Hammar et al. (81)	31 patients with First Episode (PG) 31 individually matched Healthy controls (HC)	26 (6)	15 females	14(2)	MADRS: 24 (4)	First Episode	Cross-sectional	Experimental Paradigm based on visual attention	First Episode patients show no impairment on an effortful visual attention task
Hammar and Årdal (82)	T1: 21 patients diagnosed with MDD	T1: 43 (10)	11 females	14 (4)	T1 HDRS: 22 (4) T2 HDRS: 6 (5)	10 (13)	Longitudinal with a 10 year follow up (T2)	Experimental Paradigm based on visual attention	Patients with recurrent MDD showed impairment at baseline, however normalized performance in a 10-year follow up
The trait hypothesis									
Lee et al. (44)	15 samples with 644 patients	39 (10)	Not reported	Not reported	Not reported	First episode patients	Meta-analysis	Digit span forwards; spatial span forwards Digit span backwards; spatial span backwards Trail Making Test B	Attention seems to be a trait-marker
Clery-Melin and Gorwood (84)	508 depressed patients	44 (13)	60% females	31% below high school	QIDS-SR: 16 (5)	First Episode: 62% 1 episode: 15% 2 and more episodes: 23%	Cross-sectional	d2 TMT	Findings indicated a stable marker of attentional deficit

*BACS, The brief assessment of cognition in schizophrenia (BACS); IL-6 = CANTAB, Cambridge Neuropsychological Tests Automated Battery; PHQ-9, Patient health questionnaire; QIDS-SR, The Self-Report Quick Inventory of Depressive Symptomatology; HDRS, Hamilton Depression Rating Scale*.

In sum, an update from the past decade on attentional deficits in depression shows that several aspects of attention are affected, both during the depressive episode and as a residual symptom. The role of attention deficits in relapse and development of new episodes is still unclear and impairments in this domain probably influence results in the other domains (35).

### The Domain of Processing Speed

Sometimes patients with previous episodes of depression state that they need more time to complete tasks compared to earlier, this is something we often define as processing speed, psychomotor tempo or information processing. In the clinical setting, it could be labeled latency time and can be quite severe in some severely depressed in-patients (42). Processing speed is consistently impaired in the acute phases of MDD (33; 54) thus supporting a state perspective. Pu et al. (46) however, found only minor relationships between motor-speed and depressive symptoms. Zhang et al. (86) also found slower improvements in processing speed rather than depressive symptoms, suggesting persisting deficits. Albert et al. (36) found a composite measure of processing speed to be the most impaired in MDD. Similarly to EF above, the authors find an interaction between age, duration of depression, reduced processing speed, although current symptoms of depression did not influence this processing speed (when controlling for age, race, sex, educational level and medical comorbidity), thus supporting the trait and scar hypothesis. Meluken et al. (55) did not find deficits in processing speed in relatively young MDD population and related twins, in contrast to state and trait perspectives. The study from Saleh et al. (76) suggest that early traumatic experiences could influence processing speed in MDD, and Chakrabarty et al. (77) found that a MDD population with trauma showed persisting deficits in processing speed following remission. In addition, Jaeger (87), in a review of the digit symbol substitution test, i.e., a measure of processing speed, cites research that finds consistent impairments in processing speed and effect sizes that increase in elderly MDD populations. This is supported in Boedeker et al. (74), who found deficits in an elderly MDD population. Other studies have shown that patients in remission of recurrent depression also suffer from impairment in processing speed (47, 51, 53). Xu et al. (47) did, however, find the most substantial improvements on measures of processing speed. This is in line with Schmid (30, 62). Egerhazi et al. (52) also observed an improvement in psychomotor speed during remission. Vasavada et al. (54) found improvements only in processing speed. Meta studies support this: Patients with first-episode MDD showed an impairment in psychomotor speed in the depressive state and the authors concluded that this deficit was associated with clinical state (44). Ahern and Semkovska (45) also reported in their review and meta-analysis of first-episode depressed patients that remission was associated with normalization of function in processing speed. However, different subgroups could show different impairments (46) (See Table 4).

**Table 4:** Findings within the domain of processing speed regarding origin of impairment.

**Study**	***N***	**Age (SD)**	**Sex**	**Education**	**Depression severity**	**Number of episodes**	**Study design**	**Neuropsychological tests**	**Key outcomes**
The state hypothesis									
Lee et al. (44)	15 samples with 644 patients	39 (10)	Not reported	Not reported	Not reported	First episode patients	Meta-analysis	Trail Making Test A; Digit Symbol-Coding; Symbol Digit Modalities Test	Psychomotor speed was associated with clinical state
Egerhazi et al. (52)	25 patients in acute phase (AP) 11 patients re-tested in remitted phase (RP)	AP: 57 (8) RP: 55 (6)	AP: 14 females RP: 9 females	Not reported	AP HDRS: 23 (5) RP: HDRS: 8(4)	Not reported	Longitudinal Baseline and 6 months follow up	CANTAB	Cognitive impairment is mood related with an improvement in psychomotor speed during remission
Vasavada et al. (54)	44 MDD 33 demographically similar controls (CG)	MDD: 41 (13) CG: 39 (12)	MDD: 26 females CG: 19 females	MDD: 16 (3) CG: 17 (2)	MADRS T1 = 37 (8) T4 = 17 (12)	>1 Episode, 16 years mean duration	Longitudenal	Trail A, Digit span	Processing speed only domain improving
Ahern and Semkovska (45)	31 studies with 994 patients	Weighted mean age: Patient 27 Control 30	patients: 586 females Control: 761 females	Not reported	Not reported	First Episode patients	Review and meta-analysis	TMTA, number-coding, symbol digit- modalities, substitution test, Stroop I/II,	Remission was associated with a normalization of function in processing speed
Jaeger (87)	Review of specific studies using the digit symbol substitution task	Not reported	Not reported	Not reported	Not reported	Not reported	Review	digit symbol substitution task	Consistently impaired performance on the digit symbol substitution task
Mak et al. (71)	35 MDD 35 Healthy matched controls (hMC)	MDD: 25 (4) hMC: 22 (3)	20 MDD 23 females hMC	MDD: 14 (2) hMC: 15 (1)	MADRS MDD: 23 (5)	1 (1)	Cross sectional case-control	TMT	MDD scored worse than hMC on processing speed
Pu et al. (46)	170 patients with non-psychotic MDD	38 (12)	79 females	Duration of education: 15 (2)	HAMD: 8 (4)	Not reported Duration of illness 8 (6) years	Cross-sectional	Brief Assessment of Cognition in Schizophrenia (BACS) Verbal memory: List Learning Test	A subgroup with MDD showed processing speed deficits
The scar hypothesis									
Saleh et al. (76)	64 antidepressant free depressed (PG) 65 non depressed (CG)	PG: 35(9) CG: 29(9)	39 females PG 43 females CG	PG: 35.1(8.9) CG:29(9.2)	MADRS PG: 25(5)	Episodes not reported, duration in years 6 (5)	Cross sectional case-control	Composite consisiting of TMTA, Stroop 1, symbol digit modalities	Early traumatic experiences could influence processing speed in MDD
Chakrabarty et al. (77)	MDD without maltreatment (DM+): 93 MDD with maltreatment (DM-): 90 Healthy controls with maltreatment (HM+): 22 Healthy controls without maltreatment (HM-): 80	DM+: 37 (12) DM-:34 (13) HM+: 34 (10) HM-: 33 (11)	DM+: 63 females DM-: 51 females HM+: 12 females HM-:54 females	DM+: 14 (2) DM-: 14(2) HM+: 16 (2) HM-: 16 (2)	MADRS DM+: 31 (6) DM-: 29 (6)	DM+: 4 (4) DM-:3 (3)	Longitudinal with baseline, 8 weeks and 16 weeks follow-up	Central Nervous System Vital Signs (CNS-VS) computerized battery with a global composite score	Maltreatment may be a risk factor for more severe and persistent cognitive deficits in adult MDD
Semkovska et al. (14)	11 882 major depressive episode remitters 8,533 healthy controls	Not reported specifically	Not reported specifically	Not reported specifically	Not reported specifically	Not reported specifically	Systematic review and meta-analysis	TMT A, Digit symbol Test	Number of episodes showed significant relationship to digits symbol (largest) and TMT A
The trait hypothesis									
Wekking et al. (53)	137 remitted MDD patients	45 (9)	102 females	14 (2)	HDRS: 4 (23)	6 (9)	Cross-sectional	Stroop I (Color) Stroop II (Word)	Persisting PS deficits unrelated to prior course of illness (except age of onset)
Xu et al. (47)	293 Unipolar depression patients (UP) 202 Healthy Controls (HC)	UP: 35 (13) HC:34 (10)	162 females	UP: 11 (4) HC: 13 (4)	HDRS: 27 (6)	2 (2)	Longitudinal Baseline and 6 weeks follow up	Processing speed: Trail Marking Test-part A (TMT-A) Digit symbol of Wechsler Adult Intelligence Scale	Remitted unipolar patients showed cognitive impairment in processing speed
Shimizu et al. (51)	43 remitted MDD patients (PG) 43 healthy Controls (HC)	PG: 38 (9) HC: 39 (11)	PG: 10 females HC 18 females	PG: 15 (2) HC: 15 (1)	HAM-D: 3 (2)	2 (1)	Cross-sectional	Continuous performance test (CPT) Trail Marking Test (TMT)	Patients in remission of recurrent depression show impairment I processing speed
Albert et al. (36)	91 depressed (PG) 105 non-depressed (HC)	PG: 36(9) HC:30(9)	PG: 61 females HC: 68 females	PG: 15 (2) HC: 16 (2)	MADRS: 24 (4)	Mean Duration in days: 2,116 (1,800)	Cross-sectional	Processing speed: Symbol–Digit Modality Trail Making A Stroop Color Naming condition	Found a composite measure of processing speed to be the most impaired in MDD

*MADRS, Montgomery-Åsberg Depression Rating Scale*.

In sum, processing speed seems to be the most impaired aspect of cognition in depression, but is also most influenced by state trait (and scar) effects. Subgroups in MDD could show more impairment. Results regarding processing speed in MDD deviate in several instances; however, altogether, it seems that recurrent patients show a prevalent slowing in processing speed, whereas first-episode patients show normalization of speed in remission. This pattern might indicate a scaring effect on speed, but also effects of increased aging.

## Discussion

The recent literature regarding cognitive impairment and neurocognitive profiles in MDD shows various and divergent results. There are findings of impaired cognitive functioning across domains in a long-term perspective. All three hypotheses regarding neuropsychological profiles; state, scar and trait receive various degrees of support. More specifically, while the neurocognitive profile in the attention and memory domains is more unclear, particular aspects in the EF domain, such as inhibition (and switching?), seem to show a trait-related neurocognitive profile and could contribute to the vulnerability toward relapse and recurrent episode. Further, processing speed seems to be best explained as a result of a scarring effect. Another conclusion, drawn from the current review is that it seems that the state related neurocognitive profile is more evident in patients with their first episode in MDD; such a conclusion will support a scar profile over time related to duration and number of episodes.

Most studies show cognitive impairment in most domains [(2); Snyder, Semkovska]; however, some studies report non-findings, where the patient group shows intact functioning across domains (38, 81). These reports are fewer in number, probably because science has a tradition of publishing group differences rather than null finings. Semkovska et al. (14) did not find evidence for bias in most of their included variables.

Still, many patients with a prior history of depression report that they struggle with everyday cognition, such as organizing activities, maintaining attention during a conversation and being more vulnerable to distractions in crowded spaces. They indicate that these challenges lead to stress and feelings of being unable to satisfy their own, or others' expectations. This may create an interpersonal vulnerability. Self-report measures reveal these subjective cognitive problems to a much larger degree than measures with objective standardized tests or experimental paradigms (31, 33). Although the patients have many of their cognitive skills intact, one might wonder why they still struggle to use them optimally in everyday life and thereby underestimate their actual cognitive potential due to negative bias and depressive residual symptoms. If the cognitive capacity is limited in one area, this will have an impact on other areas, since daily life functioning requires multiple simultaneous cognitive skills to enable a person to function optimally. In addition, depressive biases could influence self-report and contribute to negative self-ratings, which could explain why symptoms and self-reported cognition and depressive symptoms show greater relationships than self-report and neuropsychological results (88, 89). Reduced cognitive functioning in phases of recovery and being unable to achieve at a premorbid level may lead to negative self-representation and ruminative tendencies (7, 90, 91), and thus increase the risk of relapse and recurrence of the illness (29, 38, 92). Self-reported cognitive deficits are related to both functional disability outcome and are predictors of relapse and recurrent episodes (31, 33).

### Targeted Treatment for Cognitive Residual Symptoms

Because of the association between cognitive residual symptoms and the risk of relapse and new episodes, we have to invent treatment programmes (93) targeting these symptoms both acutely (94), and in remission (42, 95). By targeting the cognitive residual symptoms with interventions that remediate or enhance the cognitive capacity one might prevent the negative loop as consequence of failing to function optimally in everyday life (96, 97).

Cognitive enhancement therapy (CET) comprises three important elements; (1) psychoeducation of cognitive residual symptoms, (2) strategies and training of cognitive residual symptoms, (3) transfer the skills to everyday life functioning (96). There are however, several major challenges that must be addressed before such interventions can be standardized treatments of cognitive residual symptoms. First, knowledge regarding cognitive residual symptoms has been acknowledge and understood among healthcare personal and has to be incorporated both in education and in therapist training. Secondly, the frontiers of such interventions have to be explored in open and full trials with specified outcome measures, with a clear goal of enhancing the cognitive capacity in this patient group with a transfer to everyday life functioning. Thirdly, such interventions must be available to the patient group, which is normally outside primary care, since end of treatment for depression is often set at remission of mood symptoms. One way to achieve this is to make CET available in e-health care. Conclusions from a recent open pilot study of an internet-delivered CET intervention showed high compliance and feasibility in such an approach, besides the fact that the remitted MDD patients reported significantly less cognitive residual symptoms after the intervention and that this improvement prevailed at 6 months' follow-up (98). Through in-depth knowledge regarding neurocognitive profiles of depression, it will be possible to target specific aspects in CET treatment to prevent chronic course, disability, and potentially reduce the incidence of dementia. Recent and high-quality evidence on the effectiveness of cognitive-oriented psychosocial interventions has been provided in the treatment of other mental disorders characterized by cognitive impairment, e.g., schizophrenia (99) and one might expect that these promising findings may also be applicable for remitted MDD patients with cognitive residual symptoms.

### Limitations

It is important to note that the present study is not a systematic review. It is based on a comprehensive literature search and is intended to present a narrative review to identify research gaps in the field and highlight methodological concerns. This, however, comes with the risk of not being able to clarify issues such as the future research questions that are not needed (100). Moreover, this summary has not found any cohort studies measuring cognitive functioning prior to first episode of depression, which is the ideal design for support of the trait hypothesis. Following this, the presented literature supporting the trait hypothesis should be interpreted as tentative.

## Conclusions and Future Studies

MDD is characterized by residual cognitive symptoms. The origin of these residual symptoms can be explained by three major neurocognitive profiles: the scar profile, the state profile and the trait profile. However, the understanding of the origin and role in the neurocognitive profile is still oversimplified, and further knowledge is needed in order to enhance our understanding of the complexity of cognitive impairment in depression.

We therefore suggest a shift of focus in two main areas when studying the neurocognitive profile in depression: (1) A shift in focus from domain level to aspect level in cognitive functioning (see Figure 2). As an example, studying EF at the domain level might provide general and unspecific knowledge, with the risk of concluding intact EF functions in people with a history of depression. In contrast, when focusing at an aspect level, such as inhibition in EF, it is evident that this provides a more nuanced knowledge regarding the role and origin of neurocognitive profiles in depression. (2) A shift in focus from considering that depression labels one unitary group with little or no differentiation with regard to age, onset, duration, number of episodes, etc., to a much more nuanced diagnostic approach. In addition, we suggest a focus on possible origins to the onset of depression (such as inheritance or life events), when including patients in future studies. We expect that an in-depth, careful analysis of patients prior to inclusion, as opposed to the understanding of depression as a unitary group, will contribute toward discovering subgroups of patients with neurocognitive profiles more prone to lead to cognitive residual symptoms.

Defining the neurocognitive profiles in depression could have significant consequences when developing new treatments targeting cognitive residual symptoms so as to prevent relapse, new episodes and increased the risk of neurodegenerative disorders later in life.

## Author Contributions

All authors have contributed to the writing of the Introduction, Methodological, Result and Discussion Sections. ÅH and EHR have been responsible for the tables and ÅH for the figures. All authors have approved the final manuscript.

## Funding

The article is funded by the University of Bergen.

## Conflict of Interest

The authors declare that the research was conducted in the absence of any commercial or financial relationships that could be construed as a potential conflict of interest.

## Publisher's Note

All claims expressed in this article are solely those of the authors and do not necessarily represent those of their affiliated organizations, or those of the publisher, the editors and the reviewers. Any product that may be evaluated in this article, or claim that may be made by its manufacturer, is not guaranteed or endorsed by the publisher.

## References

[1] Douglas KM Porter RJ. Longitudinal assessment of neuropsychological function in major depression. Austral N Zeal J Psychiatry. (2009) 43:1105–17. 10.3109/00048670903279887 20001409

[2] Hammar Å Årdal G. Cognitive functioning in major depression – a summary. Front Hum Neurosci. (2009) 3:26. 10.3389/neuro.09.026.2009 19826496 PMC2759342

[3] Goodall J Fisher C Hetrick S Phillips L Parrish EM Allott K. Neurocognitive functioning in depressed young people: a systematic review and meta-analysis. Neuropsychol Rev. (2018) 28:216–31. 10.1007/s11065-018-9373-9 29680959

[4] Tran T Milanovic M Holshausen K Bowie CR. What is normal cognition in depression? Prevalence and functional correlates of normative versus idiographic cognitive impairment. Neuropsychology. (2021) 35:33. 10.1037/neu0000717 33393798

[5] Wagner S Doering B Helmreich I Lieb K Tadic A. A meta-analysis of executive dysfunctions in unipolar major depressive disorder without psychotic symptoms and their changes during antidepressant treatment. Acta Psychiatr Scand. (2012) 125:281–92. 10.1111/j.1600-0447.2011.01762.x 22007857

[6] Douglas KM Gallagher P Robinson LJ Carter JD McIntosh VV Frampton CM. Prevalence of cognitive impairment in major depression and bipolar disorder *Bipolar Disord*. (2018) 20:260–74. 10.1111/bdi.12602 29345037

[7] Snyder HR Miyake A Hankin BL. Advancing understanding of executive function impairments and psychopathology: bridging the gap between clinical and cognitive approaches. Front Psychol. (2015) 6:328. 10.3389/fpsyg.2015.00328 25859234 PMC4374537

[8] Clark M DiBenedetti D Perez V. Cognitive dysfunction and work productivity in major depressive disorder. Expert Rev Pharmacoecon Outcomes Res. (2016) 16:455–63. 10.1080/14737167.2016.1195688 27268275

[9] Gupta M Holshausen K Best MW Jokic R Milev R Bernard T. Relationships among neurocognition, symptoms, and functioning in treatment-resistant depression. Arch Clin Neuropsychol. (2013) 28:272–81. 10.1093/arclin/act002 23343778

[10] Woo YS Rosenblat JD Kakar R Bahk WM McIntyre RS. Cognitive deficits as a mediator of poor occupational function in remitted major depressive disorder patients. Clin Psychopharmacol Neurosci. (2016) 14:1–16. 10.9758/cpn.2016.14.1.1 26792035 PMC4730927

[11] Årdal G Lund A Hammar Å. Health-related quality of life in recurrent major depressive disorder-a 10-year follow-up study. Nord J Psychiatry. (2012) 67:339–43. 23245634 10.3109/08039488.2012.746730

[12] Sumiyoshi T Watanabe K Noto S Sakamoto S Moriguchi Y Hammer-Helmich L. Relationship of subjective cognitive impairment with psychosocial function and relapse of depressive symptoms in patients with major depressive disorder: analysis of longitudinal data from perform-j. Neuropsychiatr Dis Treat. (2021) 17:945–55. 10.2147/NDT.S288108 33814911 PMC8009536

[13] Årdal G Hammar Å. Is impairment in cognitive inhibition in the acute phase of Major Depression irreversible? Results from a 10 year follow up study. Psychol Psychother Theory Res Pract. (2011) 84:141–50. 10.1348/147608310X502328 22903853

[14] Semkovska M Quinlivan L O'Grady T Johnson R Collins A O'Connor J. Cognitive function following a major depressive episode: a systematic review and meta-analysis. Lancet Psychiatry. (2019) 6:851–61. 10.1016/S2215-0366(19)30291-3 31422920

[15] Dotson VM McClintock SM Verhaeghen P Kim JU Draheim AA Syzmkowicz SM. Depression and cognitive control across the lifespan: a systematic review and meta-analysis. Neuropsychol Rev. (2020) 30:461–76. 10.1007/s11065-020-09436-6 32385756 PMC9637269

[16] Ahern E Bockting CL Semkovska M. A hot-cold cognitive model of depression: integrating the neuropsychological approach into the cognitive theory framework. Clin Psychol Eur. (2019) 1:1–35. 10.32872/cpe.v1i3.34396

[17] Allott K Fisher CA Amminger GP Goodall J Hetrick S. Characterizing neurocognitive impairment in young people with major depression: state, trait, or scar? Brain Behav. (2016) 6:e00527. 10.1002/brb3.527 27781141 PMC5064339

[18] Hasselbalch BJ Knorr U Kessing LV. Cognitive impairment in the remitted state of unipolar depressive disorder: a systematic review. J Affect Disord. (2011) 134:20–31. 10.1016/j.jad.2010.11.011 21163534

[19] Peters AT Jacobs RH Crane NA Ryan KA Weisenbach SL Ajilore O. Domain-specific impairment in cognitive control among remitted youth with a history of major depression. Early Interv Psychiatry. (2017) 11:383–92. 10.1111/eip.12253 26177674 PMC4844809

[20] Gorwood P Corruble E Falissard B Goodwin GM. Toxic effects of depression on brain function: impairment of delayed recall and the cumulative length of depressive disorder in a large sample of depressed outpatients. Am J Psychiatry. (2008) 165:731–9. 10.1176/appi.ajp.2008.07040574 18381906

[21] Moylan S Maes M Wray NR Berk M. The neuroprogressive nature of major depressive disorder : pathways to disease evolution and resistance, and therapeutic implications. Mol Psychiatry. (2012) 18:595–606. 10.1038/mp.2012.33 22525486

[22] Frank E Prien RF Jarrett RB Keller MB Kupfer DJ Lavori PW. Conceptualization and rationale for consensus definitions of terms in major depressive disorder: remission, recovery, relapse, and recurrence. Arch Gen Psychiatry. (1991) 48:851–5. 10.1001/archpsyc.1991.01810330075011 1929776

[23] Cowen PJ. Neuroendocrine and neurochemical processes in depression. In: DeRubeis RJ, Strunk DR, editors. The Oxford Handbook of Mood Disorders. Oxford: Oxford University Press (2015), p. 190–200. 10.1093/oxfordhb/9780199973965.013.17

[24] Hansson P Murison R Lund A Hammar Å. Cognitive functioning and cortisol supression in recurrent major depression. PsyCh J. (2013) 2:167–74. 10.1002/pchj.29 26271361

[25] Hansson P Murison R Lund A Hammar Å. Cognitive functioning and cortisol in first episode major depression. Scand J Psychol. (2015) 56:379–83. 10.1111/sjop.12230 26032571

[26] Vreeburg SA Hoogendijk WJ van Pelt J DeRijk RH Verhagen JC van Dyck R. Major depressive disorder and hypothalamic-pituitary-adrenal axis activity: results from a large cohort study. Arch Gen Psychiatry. (2009) 66:617–26. 10.1001/archgenpsychiatry.2009.50 19487626

[27] Hardeveld F Spijker J De Graf R Nolen WA Beekman A. Prevalence and predictors of recurrence of major depressive disorder in the adult population. Acta Psychiatr Scand. (2010) 122:184–91. 10.1111/j.1600-0447.2009.01519.x 20003092

[28] Kessler RC Bromet EJ. The epidemiology of depression across cultures. Annu Rev Public Health. (2013) 34:119–38. 10.1146/annurev-publhealth-031912-114409 23514317 PMC4100461

[29] Majer M Ising M Künzel H Binder EB Holsboer F Modell S. Impaired divided attention predicts delayed response and risk to relapse in subjects with depressive disorders. Psychol Med. (2004) 34:1453–63. 10.1017/S0033291704002697 15724876

[30] Schmid M Hammar Å. A follow-up study of first episode major depressive disorder. Impairment in inhibition and semantic fluency – potential predictors for relapse? Front Psychol. (2013) 4:633. 10.3389/fpsyg.2013.00633 24062714 PMC3772336

[31] Manit S Ming MY Kuang YY Herng-Nieng C. Cognitive dysfunction in asian patients with depression (cogDAD): a cross-sectional study. Clin Pract Epidemiol Mental Health. (2017) 13:185. 10.2174/1745017901713010185 29238395 PMC5712642

[32] Schmid M Hammar Å. First-episode patients report cognitive difficulties in executive functioning 1 year after initial episode of major depressive disorder. Front Psychiatry. (2021) 12:667238. 10.3389/fpsyt.2021.667238 34135786 PMC8200526

[33] Saragoussi D Touya M Haro JM Jönsson B Knapp M Botrel B. Factors associated with failure to achieve remission and with relapse after remission in patients with major depressive disorder in the PERFORM study. Neuropsychiatr Dis Treat. (2017) 13:2151. 10.2147/NDT.S136343 28860772 PMC5558880

[34] Snyder HR. Major depressive disorder is associated with broad impairments on neuropsychological measures of executive function: a meta-analysis and review. Psychol Bull. (2013) 139:81–132. 10.1037/a0028727 22642228 PMC3436964

[35] Porter RJ Robinson LJ Malhi GS Gallagher P. The neurocognitive profile of mood disorders-a review of the evidence and methodological issues. Bipolar Disord. (2015) 17:21–40. 10.1111/bdi.12342 26688288

[36] Albert KM Potter GG McQuoid DR Taylor WD. Cognitive performance in antidepressant-free recurrent major depressive disorder. Depress Anxiety. (2018) 35:694–9. 10.1002/da.22747 29637661 PMC6105441

[37] Dybedal GS Tanum L Sundet K Gaarden TL Bjølseth TM. Neuropsychological functioning in late-life depression. Front Psychol. (2013) 4:381. 10.3389/fpsyg.2013.00381 23818887 PMC3694218

[38] Aker M Harmer C Landrø II. More rumination and less effective emotion regulation in previously depressed women with preserved executive functions. BMC Psychiatry. (2014) 14:334. 10.1186/s12888-014-0334-4 25427967 PMC4253635

[39] Raudeberg R Iverson GL Hammar Å. Norms matter : U. S. normative data under- estimate cognitive deficits in Norwegians with schizophrenia spectrum disorders. Clin Neuropsychol. (2019) 33:58–74. 10.1080/13854046.2019.1590641 30957642

[40] Parkinson WL Rehman Y Rathbone M Upadhye S. Performances on individual neurocognitive tests by people experiencing a current major depression episode: a systematic review and meta-analysis. J Affect Disord. (2020) 276:249–59. 10.1016/j.jad.2020.07.036 32697706

[41] Morey-Nase C Phillips LJ Bryce S Hetrick S Wright AL Caruana E. Subjective experiences of neurocognitive functioning in young people with major depression. BMC Psychiatry. (2019) 19:209. 10.1186/s12888-019-2197-1 31272419 PMC6609361

[42] Myklebost SB Amundsen OM Geraghty AWA Lnal Y Hammar A Nordgreen T. Developing an internet-delivered intervention targeting residual cognitive symptoms after major depressive disorder: a person-based approach. J Ment Health. (2022). 10.1080/09638237.2021.2022618. [Epub ahead of print]. 34983282

[43] Joormann J Gotlib IH. Emotion regulation in depression: relation to cognitive inhibition. Cogn Emot. (2010) 24:281–98. 10.1080/02699930903407948 20300538 PMC2839199

[44] Lee RS Hermens DF Porter MA Redoblado-Hodge MA. A meta-analysis of cognitive deficits in first-episode major depressive disorder. J Affect Disord. (2012) 140:113–24. 10.1016/j.jad.2011.10.023 22088608

[45] Ahern E Semkovska M. Cognitive functioning in the first-episode of major depressive disorder: a systematic review and meta-analysis. Neuropsychology. (2017) 31:52–72. 10.1037/neu0000319.supp 27732039

[46] Pu S Noda T Setoyama S Nakagome K. Empirical evidence for discrete neurocognitive subgroups in patients with non-psychotic major depressive disorder: clinical implications. Psychol Med. (2018) 48:2717–29. 10.1017/S003329171800034X 29679991

[47] Xu G Lin K Rao D Dang Y Ouyang H Guo Y. Neuropsychological performance in bipolar I, bipolar II and unipolar depression patients: a longitudinal, naturalistic study. J Affect Disord. (2012) 136:328–39. 10.1016/j.jad.2011.11.029 22169253

[48] Hammar Å Schmid M. Visual memory performance in patients with major depression: a 9-month follow-up. Appl Neuropsychol Adult. (2013) 20:192–6. 10.1080/09084282.2012.670170 23413780

[49] Hammar Å Isaksen L Schmid M Årdal G Strand M. MDD patients show intact memory performance when given optimal conditions. Appl Neuropsychol. (2011) 18:191–6. 10.1080/09084282.2011.595445 21846218

[50] Hammar Å Årdal G. Verbal memory functioning in recurrent depression during partial remission and remission, *Front Psychol*.(2013) 4:665. 10.3389/fpsyg.2013.00652 24115937 PMC3792365

[51] Shimizu Y Kitagawa N Mitsui N Fujii Y Toyomaki A Hashimoto N. Neurocognitive impairments and quality of life in unemployed patients with remitted major depressive disorder. Psychiatry Res. (2013) 210:913–8. 10.1016/j.psychres.2013.08.030 24041752

[52] Egerhazi A Balla P Ritzl A Varga Z Frecska E Berecz R. Automated neuropsychological test battery in depression - preliminary data. Neuropsychopharmacol Hungarica. (2013) 15:5–11. 23542754

[53] Wekking EM Bockting CL Koeter MW Schene AH. Cognitive functioning in euthymic recurrently depressed patients: relationship with future relapses and prior course of disease. J Affect Disord. (2012) 141:300–7. 10.1016/j.jad.2012.03.034 22613451

[54] Vasavada MM Leaver AM Njau S Joshi SH Ercoli L Hellemann G. Short- and long-term cognitive outcomes in patients with major depression treated with electroconvulsive therapy. J ECT. (2017) 33:278–85. 10.1097/YCT.0000000000000426 28617690 PMC5705261

[55] Meluken I Ottesen NM Harmer CJ Scheike T Kessing LV Vinberg M. Is aberrant affective cognition an endophenotype for affective disorders? A monozygotic twin study. Psychol Med. (2019) 49:987–96. 10.1017/S0033291718001642 29962367

[56] Mackenzie LE Uher R Pavlova B. Cognitive performance in first-degree relatives of individuals with vs without major depressive disorder: a meta-analysis. JAMA Psychiatry. (2019) 76:297–305. 10.1001/jamapsychiatry.2018.3672 30586133 PMC6439825

[57] Tully PJ Debette S Tzourio C. The association between systolic blood pressure variability with depression, cognitive decline and white matter hyperintensities: the 3C Dijon MRI study. Psychol Med. (2018) 48:1444–53. 10.1017/S0033291717002756 28950920

[58] McIntyre RS Lee Y Carmona NE Subramaniapillai M Cha DS Lee J. Characterizing, assessing, and treating cognitive dysfunction in major depressive disorder. Harv Rev Psychiatry. (2018) 26:241–9. 10.1097/HRP.0000000000000171 30188336

[59] Javaherian K Newman BM Weng H Hassenstab J Xiong C Coble D. Examining the complicated relationship between depressive symptoms and cognitive impairment in preclinical Alzheimer disease. Alzheimer Dis Assoc Disord. (2019) 33:15–20. 10.1097/WAD.0000000000000284 30489279 PMC6389418

[60] Bora E Harrison BJ Yücel M Pantelis C. Cognitive impairment in euthymic major depressive disorder: a meta-analysis. Psychol Med. (2013) 43:2017–26. 10.1017/S0033291712002085 23098294

[61] Miyake A Friedman NP Emerson MJ Witzki AH Howerter A Wager TD. The unity and diversity of executive functions and their contributions to complex frontal lobe tasks: a latent variable analysis. Cogn Psychol. (2000) 41:49–100. 10.1006/cogp.1999.0734 10945922

[62] Schmid M Hammar Å. Cognitive function in first episode major depressive disorder: poor inhibition and semantic fluency performance. Cogn Neuropsychiatry. (2013) 18:515–30. 10.1080/13546805.2012.754748 23368851

[63] Ronold EH Schmid MT Oedegaard KJ Hammar Å. A longitudinal 5-year follow-up study of cognitive function after first episode major depressive disorder: exploring state, scar and trait effects. Front Psychiatry. (2020) 11:1395. 10.3389/fpsyt.2020.575867 33364989 PMC7750430

[64] Ronold E Schmid MT Hammar Å. Risk factors and cognitive deficits in first episode major depression: a five-year longitudinal study of explorative subgroups. Biol Psychiatry. (2021) 89:S131. 10.1016/j.biopsych.2021.02.338

[65] Hammar Å Sørensen L Årdal G Ødegaard K Kroken R Roness A. Enduring cognitive dysfunction in unipolar major depression: a test-retest study using the Stroop-paradigm. Scand J Psychol. (2010) 51:304–8. 10.1111/j.1467-9450.2009.00765.x 20042028

[66] Schmid M Strand M Ardal G Lund A Hammar A. Prolonged impairment in inhibition and semantic fluency in a follow-up study of recurrent major depression. Arch Clin Neuropsychol. (2011) 26:677–86. 10.1093/arclin/acr048 21700619

[67] Hammar Å Neto E., Clemo L, Hjetland GJ, Hugdahl, K, Elliott R. Striatal hypoactivation and cognitive slowing in patients with partial remitted and remitted major depression *PsyCh J*. (2016) 5:191–205. 10.1002/pchj.134 27293083

[68] Aker M Bø R Harmer C Stiles TC Landrø NI. Inhibition and response to error in remitted major depression. Psychiatry Res. (2016) 235:116–22. 10.1016/j.psychres.2015.11.038 26639650

[69] Roca M López-Navarro E Monzón S Vives M García-Toro M García-Campayo J. Cognitive impairment in remitted and non-remitted depressive patients: a follow-up comparison between first and recurrent episodes. European Neuropsychopharmacology. (2015) 25:1991–8. 10.1016/j.euroneuro.2015.07.020 26293584

[70] Koo PC Berger C Kronenberg G Bartz J Wybitul P Reis O. Combined cognitive, psychomotor and electrophysiological biomarkers in major depressive disorder. Eur Arch Psychiatry Clin Neurosci. (2019) 269:823–32. 10.1007/s00406-018-0952-9 30392042

[71] Mak ADP Lau DTY Chan AKW So SHW Leung O Wong SLY. Cognitive Impairment In Treatment-Naïve Bipolar II and Unipolar Depression. Sci Rep. (2018) 8:1–8. 10.1038/s41598-018-20295-3 29382902 PMC5789863

[72] Ji Y Li W Liu B Liu J Ju Y Wang M. Clinical characteristics of cognitive deficits in major depressive disorder: a 6-montprospective study. Rev Psiquiatr Clin. (2020) 47:101–5. 10.1590/0101-60830000000241

[73] Ziegelmayer C Hajak G Bauer A Held M Rupprecht R Trapp W. Cognitive performance under electroconvulsive therapy (ECT) in ECT-naive treatment-resistant patients with major depressive disorder. J ECT. (2017) 33:104–10. 10.1097/YCT.0000000000000385 28169947

[74] Boedeker S Schulz P Beblo T Lenz E Sammer G Kreisel S. Symbol comprehension in patients with alzheimer disease dementia, mild cognitive impairment, and major depressive disorder. Alzheimer Dis Assoc Disord. (2020) 34:85–93. 10.1097/WAD.0000000000000347 31567152

[75] Maalouf FT Brent D Clark L Tavitian L McHugh RM Sahakian BJ. Neurocognitive impairment in adolescent major depressive disorder: state vs. trait illness markers. J Affect Disord. (2011) 133:625–32. 10.1016/j.jad.2011.04.041 21620477 PMC4119611

[76] Saleh A Potter GG McQuoid DR Boyd B Turner R MacFall JR. Effects of early life stress on depression, cognitive performance and brain morphology. Psychol Med. (2017) 47:171–81. 10.1017/S0033291716002403 27682320 PMC5195852

[77] Chakrabarty T Harkness KL McInerney SJ Quilty LC Milev RV Kennedy SH. Childhood maltreatment and cognitive functioning in patients with major depressive disorder: a CAN-BIND-1 report. Psychol Med. (2020) 50:2536–47. 10.1017/S003329171900268X 31583989

[78] Bhardwaj A Wilkinson P Srivastava C Sharma M. Cognitive deficits in euthymic patients with recurrent depression. J Nerv Ment Dis. (2010) 198:513–5. 10.1097/NMD.0b013e3181e4c5ba 20611055

[79] Hasselbalch BJ Knorr U Hasselbalch SG Gade A Kessing LV. Cognitive deficits in the remitted state of unipolar depressive disorder. Neuropsychology. (2012) 26:642. 10.1037/a0029301 22823136

[80] Preiss M Kucerova H Lukavsky J Stepankova H Sos P Kawaciukova R. Cognitive deficits in the euthymic phase of unipolar depression. Psychiatry Res. (2009) 169:235–9. 10.1016/j.psychres.2008.06.042 19765829

[81] Hammar Å Kildal AB Schmid M. Information processing in first episode depression. Scand J Psychol. (2012) 53:445–9. 10.1111/sjop.12012 23170862

[82] Hammar Å Årdal G. Effortful information processing in patients with major depression - a 10-year follow-up study. Psychiatry Res. (2012) 198:420–3. 10.1016/j.psychres.2011.11.020 22445703

[83] Hammar A Lund A Hugdahl K. Long-lasting cognitive impairment in unipolar major depression: a 6- month follow-up study. Psychiatry Res. (2003) 118:189–96. 10.1016/s0165-1781(03)00075-1 12798984

[84] Cléry-Melin ML Gorwood P. A simple attention test in the acute phase of a major depressive episode is predictive of later functional remission. Depress Anxiety. (2017) 34:159–70. 10.1002/da.22575 27781337

[85] Ye G Yin GZ Tang Z Fu JL Chen J Chen SS. Association between increased serum interleukin-6 levels and sustained attention deficits in patients with major depressive disorder. Psychol Med. (2018) 48:2508–14. 10.1017/S0033291718000090 29415791

[86] Zhang BH Feng L Feng Y Xin LM Zhu XY Tan YL. The effect of cognitive impairment on the prognosis of major depressive disorder. J Nerv Ment Dis. (2020) 208:683–8. 10.1097/NMD.0000000000001180 32433202

[87] Jaeger J. Digit symbol substitution test. J Clin Psychopharmacol. (2018) 38:513–9. 10.1097/JCP.0000000000000941 30124583 PMC6291255

[88] Serra-Blasco M Torres IJ Vicent-Gil M Goldberg X Navarra-Ventura G Aguilar E. Discrepancy between objective and subjective cognition in major depressive disorder. Eur Neuropsychopharmacol. (2019) 29:46–56. 10.1016/j.euroneuro.2018.11.1104 30503099

[89] Bernhardt M Klauke S Schroder A. Longitudinal course of cognitive function across treatment in patients with MOD: a meta-analysis. J Affect Disord. (2019) 249:52–62. 10.1016/j.jad.2019.02.021 30753954

[90] Joormann J Stanton CH. Examining emotion regulation in depression: a review and future directions. Behav Res Ther. (2016) 86:35–49. 10.1016/j.brat.2016.07.007 27492851

[91] Sumner JA Griffith JW Mineka S. Examining the mechanisms of overgeneral autobiographical memory: capture and rumination, and impaired executive control. Memory. (2011) 19:169–83. 10.1080/09658211.2010.541467 21294036

[92] Ronold EH Joormann J Hammar Å. Facing recovery: emotional bias in working memory, rumination, relapse and recurrence of Major Depression- an experimental paradigm conducted five years after first episode of MDD. J Appl Neuropsychol Adult. (2019) 27:299–310. 10.1080/23279095.2018.1550406 30646773

[93] Pedersen A Küppers K Behnken A Kroker K Schöning S Baune BT. Implicit and explicit procedural learning in patients recently remitted from severe major depression. Psychiatry Res. (2009) 169:1–6. 10.1016/j.psychres.2008.06.001 19595464

[94] Legemaat AM Semkovska M Brouwer M Geurtsen GJ Burger H Denys D. Effectiveness of cognitive remediation in depression: a meta-analysis. Psychol Med. (2021) 1–18. 10.1017/s0033291721001100 PMC9811271 33849674

[95] Hammar A Semkovska M Borgen IMH Myklebost S Ronold EH Sveen T. A pilot study of cognitive remediation in remitted major depressive disorder patients. Appl Neuropsychol Adult. (2020). 10.1080/23279095.2020.1726919. [Epub ahead of print]. 32088993

[96] Douglas K Peckham A Porter R Hammar Å. Cognitive enhancement therapy for mood disorders: a new paradigm? Austral N Zeal Psychiatry. (2019) 53:1148–50. 10.1177/0004867419873711 31516027 PMC7290243

[97] Porter RJ Hammar Å Beevers CG Bowie CR Nodtvedt ØO Peckham AD. Cognitive and affective remediation training for mood disorders. Aust NZ J Psych. (2017) 51:317–9. 10.1177/0004867416678079 28343432 PMC6417792

[98] Myklebost SB Nordgreen T Hammar Å. An open pilot study of an internet-delivered intervention targeting self-perceived residual cognitive symptoms after major depressive disorder. Appl Neuropsychol Adult. (2021) 1–10. 10.1080/23279095.2021.1901706. [Epub ahead of print]. 33813984

[99] Vita A Barlati S Ceraso A Nibbio G Ariu C Deste G. Effectiveness, core elements, and moderators of response of cognitive remediation for schizophrenia: a systematic review and meta-analysis of randomized clinical trials. JAMA Psychiatry. (2021) 78:848–58. 10.1001/jamapsychiatry.2021.0620 33877289 PMC8058696

[100] Chalmers I Glasziou P. Avoidable waste in the production and reporting of research evidence. Lancet. (2009) 374:86–9. 10.1016/S0140-6736(09)60329-9 19525005

